# Patterning of Retinoic Acid Signaling and Cell Proliferation in the Hippocampus

**DOI:** 10.1002/hipo.22037

**Published:** 2012-06-11

**Authors:** Timothy Goodman, James E Crandall, Sonia E Nanescu, Loredana Quadro, Kirsty Shearer, Alexander Ross, Peter McCaffery

**Affiliations:** 1 Institute of Medical Sciences, University of AberdeenAberdeenshire, United Kingdom; 2 School of Biological Sciences, University of East AngliaNorwich, United Kingdom; 3 Department of Cell Biology, University of Massachusetts Medical SchoolWaltham, Massachusetts, USA; 4 University of Medicine and Pharmacy “Gr. T. Popa” IasiRomania; 5 Department of Food Science, Rutgers UniversityNew Brunswick, New Jersey, USA; 6 Rowett Institute of Nutrition and Health, University of AberdeenAberdeenshire, United Kingdom

**Keywords:** suprapyramidal, infrapyramidal, proliferation, subgranular zone, dentate gyrus

## Abstract

The nuclear receptor ligand retinoic acid (RA) has been identified as an endogenous regulatory factor in the hippocampus, acting on pyramidal neurons and granule neuron progenitors, but almost nothing is known about the distribution of RA itself in the hippocampus. This study describes the source of RA for the rodent hippocampus in the meninges via the key RA synthetic enzyme retinaldehyde dehydrogenase 2 (RALDH2). Diffusion of RA from the meninges potentially creates a gradient of RA across the infrapyramidal and suprapyramidal blades of the dentate gyrus, enhanced by the expression of the RA catabolic enzyme Cyp26B1 between the blades, and an infrapyramidal and suprapyramidal blade difference is evident in RA-regulated transcription. This asymmetry may contribute to some of the physiological and molecular differences between the blades, including a disparity in the rates of cell proliferation in the subgranular zone of the two blades through RA inhibition of cell proliferation. Such differences can be altered by either the application of excess RA, its effect dependent on the relative position along the septotemporal axis, or change in RA signaling through mutation of retinol binding protein, while the capacity of RA to inhibit proliferation of cells in the dentate gyrus is demonstrated using in vitro slice culture. Use of synthetic and catabolic enzymes in the hippocampus to create differing zones of RA concentration parallels the mechanisms used in the developing brain to generate patterns of RA-regulated transcription. © 2012 Wiley Periodicals, Inc.

## INTRODUCTION

Retinoic acid (RA) is a unique lipid regulator of neural progenitor proliferation and differentiation. RA's actions are mediated by its binding and activation of specific RA receptors that are members of a family of ligand-activated transcriptional regulators (Mark et al.,^2006^). This regulatory factor has the potential to promote transcription of a very large number of genes, potentially inducing, by at least a factor of two, several thousand genes (Cawley et al.,^2004^). Among its functions, RA induces neuronal differentiation in stem cells, for instance, RA induces embryonic stem cells to take on the features of pax-6 positive radial glial cells characteristic of endogenous embryonic neural progenitors (Bibel et al.,^2007^). RA also has antiproliferative effects on a wide variety of cell types and is used in the treatment of several cancers (Crowe,^2002^). However, the expression of different RA binding proteins in the cytoplasm can shuttle RA away from the RA receptors and toward PPARalpha/delta, resulting in RA promoting, rather than inhibiting, cell proliferation (Schug et al.,^2007^).

As a regulatory factor in the developing central nervous system (CNS), RA has been investigated extensively, where it functions to pattern gene expression and regulate neurogenesis (Maden,^2002^, ^2007^). As the embryo matures and neurogenesis, as well as other RA-regulated functions, decline, RA-regulated transcription becomes progressively restricted (Luo et al.,^2004^). Until recently, it has been little considered that RA may have a function in the adult brain, but although its action are localized to only a few brain regions, evidence indicates that RA influences synaptic plasticity in the hippocampus (Misner et al.,^2001^; Aoto et al.,^2008^; Chen and Napoli,^2008^) as well as regulating proliferation and neurogenesis in the three restricted regions where this takes place, the olfactory epithelium, subventricular zone (SVZ) and hippocampal subgranular zone (SGZ) (Asson-Batres et al.,^2003^; Haskell and LaMantia,^2005^; Wang et al.,^2005^; Jacobs et al.,^2006^). In the SVZ, RA promotes both proliferation and neuronal differentiation of progenitors (Haskell and LaMantia,^2005^; Wang et al.,^2005^). In the hippocampal SGZ, vitamin A was described to be necessary for neuronal differentiation and survival, but not proliferation of granule cells (Jacobs et al.,^2006^). Certainly, from analysis of the regions of RA's actions in the brain using transgenic RA reporter mice, the hippocampus is a particular hotspot for RA signaling (Misner et al.,^2001^; Crandall et al.,^2004^; Luo et al.,^2004^), with RA-regulated transcription localized predominantly in the dentate gyrus. In vitro studies indicate that the neuronal progenitors which give rise to the granule neurons directly respond to RA (Takahashi et al.,^1999^).

Although RA has the potential to act in the hippocampus, the endogenous source of RA to regulate function is unknown. In situ hybridization and immunohistochemistry studies have not shown the presence in the rodent hippocampus of the retinaldehyde dehydrogenases (RALDHs) required for RA synthesis. This report describes the pattern of RA synthesis in the mouse hippocampus. The only source of RA-synthesizing enzyme is retinaldehyde dehydrogenase 1 and 2 (RALDH 1 and 2) present in the adjacent meninges with RA diffusing from this source, potentially distributed nonhomogenously across the infrapyramidal and suprapyramidal blades of the dentate gyrus. This may differentially regulate function between the blades, and one example is explored is the rate of proliferation of neural precursors. It is demonstrated that RA can regulate proliferation of progenitors in the SGZ and the ratio of proliferation between the infrapyramidal and suprapyramidal blades can be shifted by alterations in RA signaling.

## METHODS

All mice used in this study were of age 2–3 months, unless otherwise stated. For paraformaldehyde (PFA) fixation of the brain, mice under Avertin® or pentobarbital anesthesia were intracardially perfused, flushing first with room temperature saline followed by a cold (4°C) 100 ml volume of 4% (PFA) in pH 7.2 0.1M phosphate buffer. The brains were removed from the skull, postfixed overnight in the same fixative at 4°C and sunk in 30% sucrose. All animals in the UK were kept in accordance with Home Office regulations and in the US following the guidelines laid down by the NIH (NIH Guide for the Care and Use of Laboratory Animals). Endogenous RA signaling in the mouse was determined using the RA response element (RARE)-lacZ reporter transgenic line (Rossant et al.,^1991^) that contains a tetrameric repeat of the RARβ2 RARE linked to the hsp68 minimal promoter which has been extensively used as a RA-reporter transgene. RBP^−/−^ mice were kindly given by Quadro and Blaner (Columbia University, New York) (Quadro et al.,^1999^). The mice were maintained on a regular chow diet manufactured by TestDiet (PicoLab Rodent Diet 20 #5053; Purina Mills, St. Louis, MO) from weaning. This diet provided 25.0, 12.0, and 63.0% calories from protein, fat, and carbohydrates, respectively. The diet also contained 25 IU of vitamin A/g of diet*.* A sledge microtome was used to cut frozen 40-μm coronal sections. These were used for immunohistochemistry using standard procedures with free-floating sections in meshed wells (CoStar) with fluorescent-conjugated secondary antibodies (Crandall et al.,^2000^; Palmer et al.,^2000^; Crandall et al.,^2004^). For bromodeoxyuridne (BrdU) immunostaining, sections were pretreated with 1M HCl for 30 min at 47°C and labeled with BrdU primary antibody (1:500 Accurate Scientific, NY) using an anti-rat secondary antibody (Alexafluor 546, Molecular Probes) as described previously (Palmer et al.,^2000^; Crandall et al.,^2004^). RALDH1 antibody was from Lindahl (University of South Dakota) and specificity by isoelectric focusing blot described in McCaffery et al. (McCaffery et al.,^1991^) and RALDH2 antibody was generated within our lab and specificity described in Berggren et al. (Berggren et al.,^1999^).

The RARE reporter mouse was used to quantify RA signaling in the dentate gyrus. LacZ-positive cells were quantified using Image J software and expressed as a percentage of the total number of granule cells. Quantification of absolute cell numbers was not used because the number of cells expressing the reporter can differ between individual animals due to the variability of reporter response potentially caused by an epigenetic mechanisms as previously reported (Sakai and Dräger,^2010^). To avoid bias caused by epigenetic variation, the percentage of lacZ-positive cells was expressed as a ratio between the two blades for the control/RA experiment.

To quantitate the BrdU-labeled cells in the SGZ of the dentate gyrus, a modified unbiased stereological protocol was used in which BrdU-labeled cells were counted in every 12th section at either 200× or 400× (West et al.,^1991^; Gould et al.,^1999^; Malberg et al.,^2000^). The average number of sections with hippocampi numbered 8, and the results were presented as the total number of cells in all sampled sections, averaging between three and five mice per treatment. A single investigator counted cells on coded slides. A labeled cell was defined to be in the SGZ if the cell touched or was within two cell diameters of the SGZ (Kuhn et al.,^1996^). Statistical analysis was performed using a two-tailed Student's *t* test with *P* < 0.05 considered significant. To determine differences in numbers of BrdU-labeled cells along the rostral caudal length of the forebrain every 12th section was selected based on its position within three segments of the forebrain, rostral, intermediate, and caudal. These matched the sections illustrated for the C57BL/J6 brain in the atlas from Paxinos and Franklin (2004) spanning approximate bregma values of −1.46 to −1.94, −2.06 to −2.54, and −2.70 to −3.16 mm for rostral, intermediate, and caudal regions, respectively.

Messenger RNA levels of Cyp26B1 were detected by in situ hybridization with coronal rat brain sections, sense, and antisense riboprobes corresponding to 2354–3285 bp of mouse Cyp26b1 (GeneBank accession number NM_181087) and following techniques as previously described in detail (Ross et al.,^2009^).

Organotypic hippocampal slice cultures were performed using a modified version of the interface method (Stoppini et al., 1991). Hippocampal slices were prepared from postnatal Day 14 RARE-lacZ pups. Following anesthesia and decapitation, the heads were sprayed thoroughly with 70% ethanol. The brain was removed from the skull and bisected down the midline. Sagittal slices (200 μm) were cut using a vibratome (Leica). The dissection and culture medium consisted of 50% minimum essential medium eagle, 25% heat inactivated horse serum, 25% Hank's Balanced Salt Solution, supplemented with 100 units penicillin, 100 μg streptomycin, 2 mM glutamine, 5 mg/ml glucose, and 25 mM 4-(2-hydroxyethyl)-1-piperazineethanesulfonic acid (HEPES). The hippocampus was dissected from the surrounding tissue under a microscope and transferred to a sterile 0.4-μm porous Millicell membrane (Millipore) using a glass pipette. Cultures were maintained at 34°C, 5% CO_2_ in 1 ml culture medium. Following 24 hr in culture, cultures were transferred to serum-free medium consisting of Neurobasal medium, B27 supplement minus vitamin A, 100 units penicillin, 100 μg streptomycin, 2 mM glutamine, and 5 mg/ml glucose. Following 3 days in culture, slices were treated with dimethyl sulfoxide, RA, epidermal growth factor (EGF) (20 ng/ml), or RA/EGF for 48 hr. BrdU (10 μM) was added 2 hr before fixation. Slices were fixed in 4% PFA for 10 min followed by 10 min in 100% methanol at −20°C. Following extensive washes in phosphate buffered saline (PBS), slices were blocked in 10% horse serum and 0.4% triton X for 2–3 days. Slices were incubated in anti-NeuN (1:500, Chemicon) diluted in 5% horse serum and 0.4% triton overnight followed by anti-mouse Alexa Fluor 555 (1:300, Invitrogen) overnight. Following washing, slices were fixed in 4% PFA for 10 min followed by four washes in PBS. DNA denaturation was performed by incubating slices in 1M hydrochloric acid at 47° C for 30 min. Following extensive washes, slices were incubated in anti-BrdU (1:1,000, OBT0030, Accurate) overnight followed by anti-rat secondary Alexa Fluor 488 (Invitrogen) overnight. Finally, slices were mounted and visualized under a fluorescence microscope. ImageJ was used for quantification of BrdU positive cells in cultured slices. NeuN staining was used to demarcate the boundaries of the dentate gyrus, CA3, and CA1 subregions. Alternatively, the same double labeling protocol was used to label cells with NeuroD (1:100, sc-1084, Santa Cruz) and BrdU to identify neuronal progenitors. BrdU or BrdU/NeuroD positive cells were quantified within the boundaries of each subregion using the automated cell counter within ImageJ for BrdU positive cells and manual cell counter for BrdU/NeuroD double positive cells. Cell counts were verified using randomized manual counting. For statistical analysis, one-way analysis of variance (ANOVA) statistical tests with Tukey's posthoc test were carried out or Student's *t* test, when appropriate.

## RESULTS

### Asymmetry in Retinoic Acid Synthesis in the Dentate Gyrus

**FIGURE 1:**
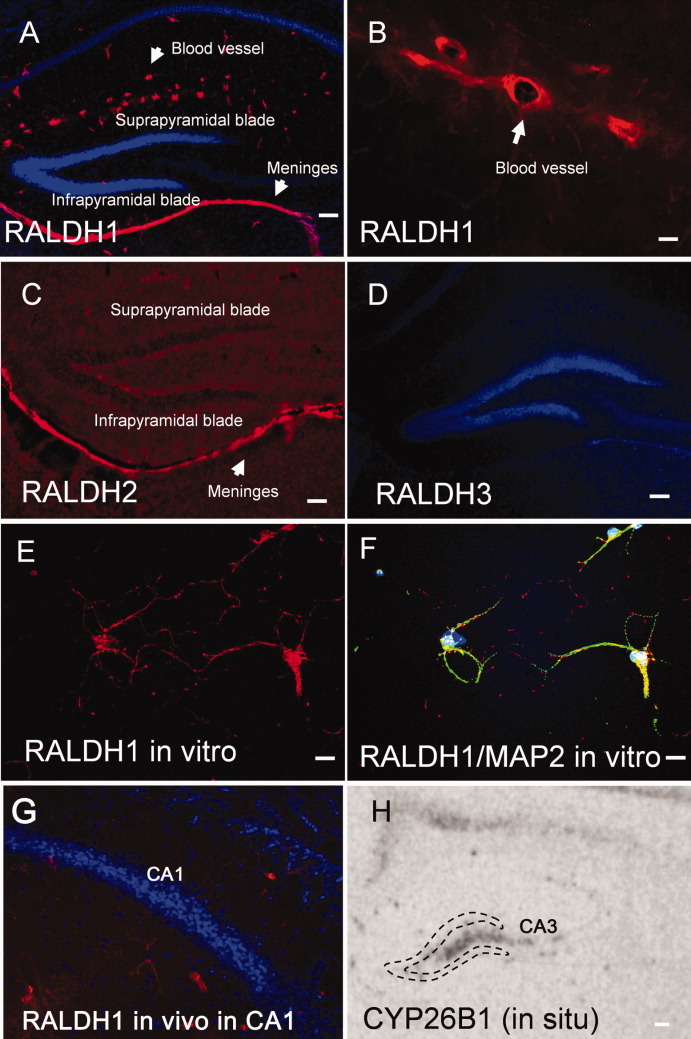
Expression of RA metabolic enzymes in the hippocampus. The RA synthetic enzymes RALDH1 (A) and RALDH2 (C) can be detected immunohistochemically in the meninges beneath the infrapyramidal blade of the hippocampus with some blood vessels along the hippocampal fissure, at the edge of the molecular layer of the suprapyramidal blade, expressing RALDH1 (A and B). In contrast, RALDH3 is absent from the hippocampus (D). Although RALDH1 is strongly detected in cultured hippocampal cells (E) and their neuronal identity demonstrated by double labeling with MAP2 (F; RALDH1 in red, MAP2 in green), it is not present at detectable levels by immunohistochemistry in the endogenous hippocampal neurons of the dentate gyrus (A) or the hippocampal subfields (G). In the rat hippocampus, the transcript of the RA catabolic enzyme, Cyp26b1, is also expressed, present in CA3 and the hilus (H). Scale bars: A, C and D, H, 90 μm; B, 18 μm; E and F, 10 μm. [Color figure can be viewed in the online issue, which is available at wileyonlinelibrary.com.]

As a regulator of CNS development in the embryo, RA functions as a locally synthesized factor regulating transcription within discrete areas (McCaffery et al.,^2003^). Although there are many fewer regions of RA synthesis in the adult brain, there are limited regions in which the RA synthetic enzymes are present, potentially creating discrete regions of high RA (Luo et al.,^2004^). In the region of the dentate gyrus, RALDH1 and RALDH2 are the only two RA synthesizing RALDHs and are predominantly expressed in the meninges adjacent to the infrapyramidal blade of the dentate gyrus (Figs. 1A,C), although RALDH1 is also in the walls of some of the large blood vessels of the hippocampal fissure (Figs. 1A,B). The third RA synthesizing enzyme, RALDH3 is absent (Fig. 1D). These enzymes were not identified in hippocampal neurons and, although it has been previously described that RALDH1 is present in cultured hippocampal neurons (Aoto et al.,^2008^), which we verify as strongly positive for RALDH1 (Figs. 1E,F), it is not present in vivo at sufficiently high levels to be detected immunohistochemically (Figs. 1A,G). The RA catabolic enzymes, Cyp26a1 and Cyp26b1, also control the distribution of RA (McCaffery and Simons,^2007^). Cyp26b1 but not Cyp26a1 is present in the hippocampus (Abu-Abed et al.,^2002^). No antibodies exist to Cyp26b1, but in situ hybridization in the rat indicates its expression in cells of the hilus between the blades of the dentate gyrus, limiting the amount of RA from the meninges reaching the suprapyramidal blade (Fig. 1H).

**FIGURE 2:**
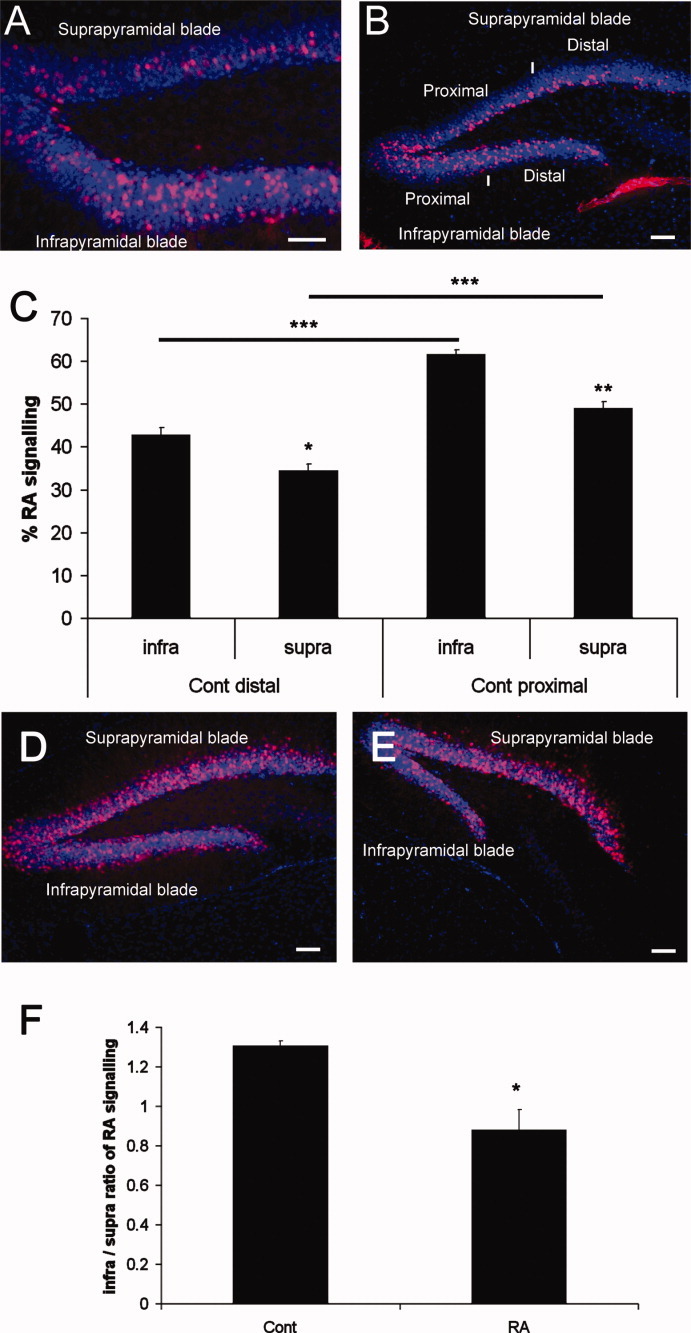
Pattern of RA-regulated transcription in the hippocampus detected using the RARE/LacZ RA reporter mouse. RA reporter gene expression in the hippocampus was detected by immunostaining for the beta-galactosidase reporter protein. A greater number of cells of the dentate gyrus are labeled in the infrapyramidal versus the suprapyramidal blade (A and B: beta-galactosidase immunostaining in red and nuclei labeled with bisbenzamide in blue). A greater number of beta-galactosidase positive cells were also evident in the proximal versus distal halves (B). When numbers of beta-galactosidase positive cells were counted in the two blades, counting separately those in the proximal and distal halves arbitrarily divided at the centre point (as shown in B), a significant difference was evident between the two blades in both proximal and distal halves (C). A significant difference was also evident between the distal and proximal portions of the infrapyramidal and suprapyramidal blade (C). When RARE/LacZ animals were injected with 30 mg/kg RA over 3 days to generate homogenous levels of RA throughout the hippocampus, this resulted in similar activation of the RA reporter in the infrapyramidal and suprapyramidal blades (D and E) significantly shifting the ratio of numbers of beta-galactosidase positive cells from its normal value of 1.3–0.9 (F). Scale bars: A, B, D, and E, 90 μm. (C) Suprapyramidal versus infrapyramidal blade in the distal part of the dentate gyrus, *n* = 3, one way ANOVA, *P* < 0.0001, Tukey's posthoc test, *P* < 0.05 (*), Suprapyramidal versus infrapyramidal blade in the proximal part of the dentate gyrus, *n* = 3, one way ANOVA, *P* < 0.0001, Tukey's posthoc test, *P* < 0.01 (**), Infrapyramidal and suprapyramidal blade in the distal dentate gyrus versus infrapyramidal and suprapyramidal blade respectively in the proximal part of the dentate gyrus, *n* = 3, one way ANOVA, *P* < 0.0001, Tukey's posthoc test, *P* < 0.001 (***). (F) Control-treated infrapyramidal/suprapyramidal blade ratio versus RA-treated infrapyramidal/suprapyramidal blade ratio, *n* = 3, Student's unpaired *t* test, *P* < 0.05 (*). [Color figure can be viewed in the online issue, which is available at wileyonlinelibrary.com.]

To determine the resulting pattern of RA-regulated transcription in the dentate gyrus, an RA reporter mouse line transgenic for a RARE coupled to a lacZ reporter gene (Rossant et al.,^1991^) was used. This allows the spatial pattern of RA signaling to be determined in a way that cannot be achieved by microdissection of tissue and physical quantification of RA owing to the low levels of RA present (Kane et al.,^2008^). Expression of RA reporter in the RARE-lacZ hippocampus was evident in a subpopulation of cells in the dentate gyrus with a greater number of beta-galactosidase positive cells in infrapyramidal compared with the suprapyramidal blade (Figs. 2A,B). To quantify this difference in RA response, numbers of positive cells were counted although, because a difference was also evident between the proximal and distal halves of each blade (evident in Fig. 2B), these two regions were counted separately. In both regions, there was a statistically significantly greater number of positive cells in the infrapyramidal than in the suprapyramidal blade (Fig. 2C).

If the preferential activation of granule cells in the infrapyramidal blades is due to the nearby source of RA generated in the meninges, this spatial pattern will be removed by systemic exposure of the RARE-lacZ mouse to RA resulting in an even distribution of RA between the two blades. Such a change in pattern to one of comparable activation of RA reporter between blades was evident with RA treatment for 3 days at 30 mg/kg (Figs. 2D,E); when numbers of beta-galactosidase positive cells were counted in the two blades of control and RA-treated RARE-lacZ mice, the ratio of positive cells between the blades significantly shifted in the RA-treated animals to approach a ratio of one (Figs. 2F).

**FIGURE 3:**
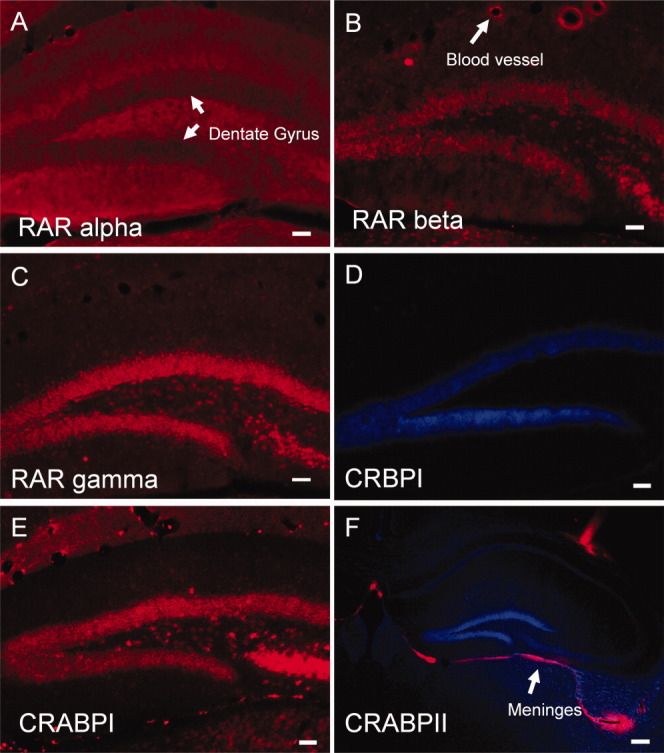
Expression of proteins transducing RA signaling in the hippocampus. RARalpha (A) was absent from the dentate gyrus whereas RARbeta (B) and RARgamma (C) were the main RA receptors present and expressed similarly between the two blades. Of the retinoid binding proteins cellular retinol binding protein I (CRBPI) was not detected (D). The cellular RA binding protein I (CRABPI) was present equally in both blades (E) whereas CRABPII was localized to the meninges below the hippocampus (F). All immunostaining used a red fluorescent second antibody and panels D and F included blue fluorescent counterstaining with bisbenzamide. Scale bars: A–E, 50 μm; F, 200 μm. [Color figure can be viewed in the online issue, which is available at wileyonlinelibrary.com.]

The difference in reporter expression between the two blades may potentially result not only from the local source of RA synthesis but also from a disparity in one or more of the proteins required for RA signal transduction. RARalpha was absent (Fig. 3A) from the dentate gyrus whereas RARbeta and RARgamma (Figs. 3B,C) were the predominant RA receptors in the dentate gyrus but were distributed evenly between the blades. The cytoplasmic RA binding protein cellular retinol binding protein I (CRBPI) was not present in the dentate gyrus (Fig. 3D), whereas cellular RA binding protein I (CRABPI), which buffers RA in the cytoplasm, was expressed evenly in both blades (Fig. 3E), whereas cellular RA binding protein II (CRABPII) was not present in the dentate gyrus but was expressed in the meninges (Fig. 3F). This suggests that the difference in RA signaling between infrapyramidal and suprapyramidal blades may be the result of a higher RA concentration around the infrapyramidal blade due to the local presence of the RA-synthesizing enzyme in the meninges, rather than a difference in another RA-signaling component.

### Control of Cell Proliferation in the Dentate Gyrus by Retinoic Acid

**FIGURE 4:**
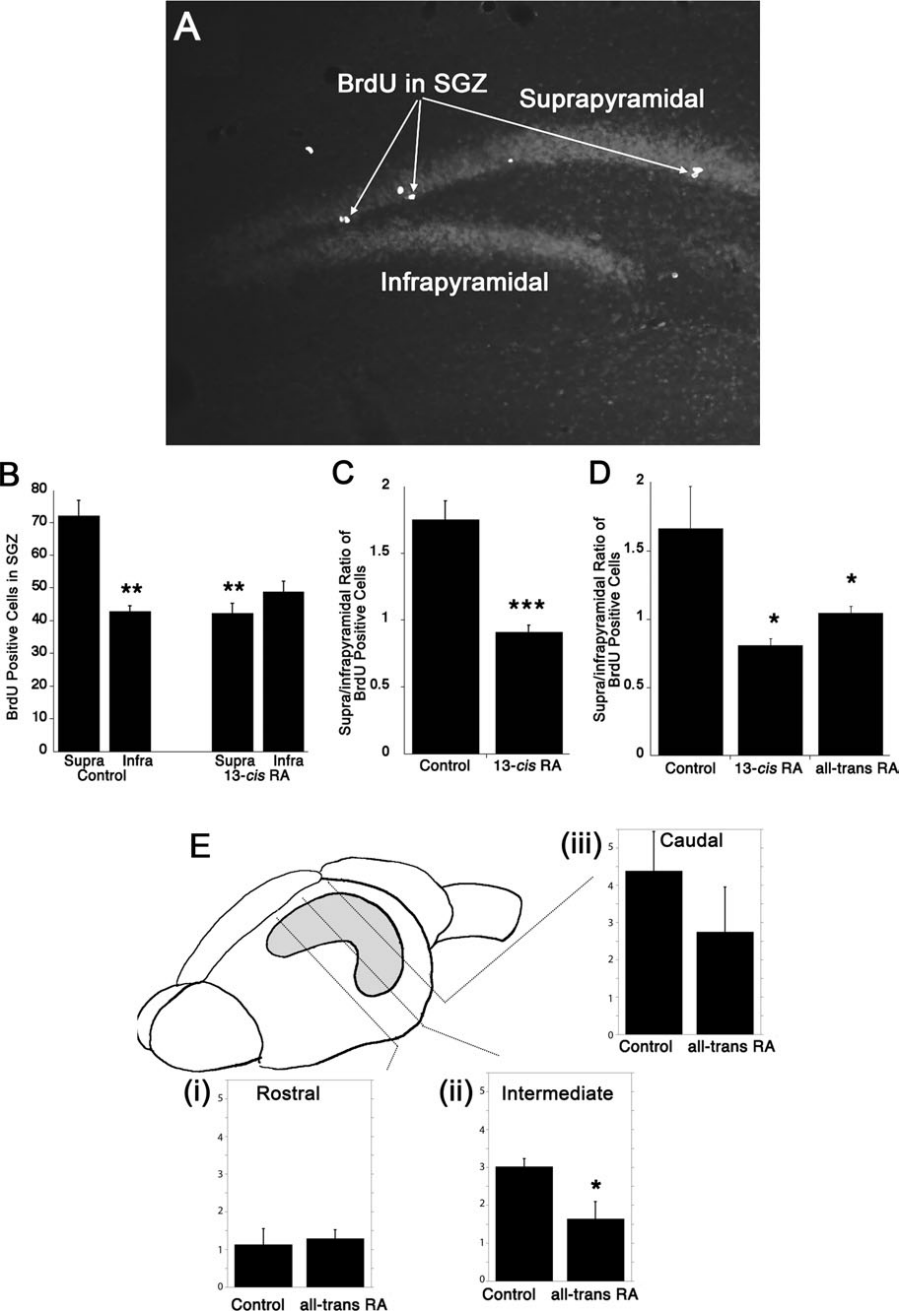
Influence of 3-week and 3-day exposure to RA on the infrapyramidal/suprapyramidal blade asymmetry on SGZ cell proliferation. A representative figure of BrdU labeling in the dentate gyrus, and greater numbers of labeled cells in the suprapyramidal compared with infrapyramidal blade, is shown (A). Control, 3-week vehicle-injected mice showed an average number of BrdU-labeled cells that was significantly higher in the suprapyramidal versus infrapyramidal blades (B, left). Treatment of mice with 13-*cis* RA over a 3-week period followed by BrdU treatment resulted in a preferential decline in average number of BrdU-labeled cells in the suprapyramidal blade that was statistically significant (B, right). When the mean ratio of BrdU-labeling in the suprapyramidal blade to infrapyramidal blade was determined, the ratio in the control-treated animals was 1.75, which significantly declined to 0.91 after chronic 13-*cis* RA treatment (C). Similarly, short-term treatment with 13-*cis* RA or all-*trans* RA over 3 days resulted in a normalization of the suprapyramidal blade to infrapyramidal blade ratio (D). The effect of all-*trans* RA to normalize the ratio of proliferating cells in the suprapyramidal versus infrapyramidal blade was examined in differing regions of the hippocampus along the rostral-caudal axis of the forebrain (E: i, ii, and iii) and found to differ markedly in each region with a normalizing influence only in the intermediate and caudal regions and significant in the intermediate area. Supra, suprapyramidal blade; Infra, infrapyramidal blade. Scale bar: A, 90 μm. (B) Control supra versus control infra, *n* = 5, one-way ANOVA, *P* < 0.01, Tukey's posthoc test, *P* < 0.01 (**), Control supra versus RA supra, one-way ANOVA, *P* < 0.01, Tukey's posthoc test, *P* < 0.01 (**). (C) control treated versus 13-cis RA treated, *n* = 5, Student's *t* test, *P* ≤ 0.0005 (***), (D) Control versus 13-*cis* RA, *n* = 5, one-way ANOVA, *P* < 0.05, Tukey's posthoc test, *P* < 0.05 (*), Control versus all-*trans* RA, *n* = 5, one-way ANOVA, *P* < 0.05, Tukey's posthoc test, *P* < 0.05 (*). (E) control treated versus all-*trans* RA treated, *n* = 3, Student's *t* test, *P* ≤ 0.015 (*).

Cell proliferation is controlled by RA in either a positive or negative manner, depending on cell type (Nabeyrat et al.,^2000^; Wakino et al.,^2001^; Paillaud et al.,^2002^; Alisi et al.,^2003^; Oliva et al.,^2003^; Ledda-Columbano et al.,^2004^). 13-*cis* RA is a “prodrug” isomer of RA removed slower from the circulation than all-*trans* RA (Khoo et al.,^1982^) but isomerized in vivo to all-*trans* RA to activate RA receptors (Tsukada et al.,^2000^). We have previously shown that a 3-week exposure to 13-*cis* RA at 1 mg/kg/day inhibits cell proliferation in the SGZ (Crandall et al.,^2004^), quantifying cell proliferation with two hourly injections of BrdU over a 6-hr period followed by sacrifice and counting of labeled cells in immunostained sections. These experiments were repeated to determine the relative influence on the two blades of the dentate gyrus. Under control conditions, when cell proliferation was compared between the infrapyramidal and suprapyramidal blades of the dentate gyrus, a significant difference was evident between the blades with lower proliferation in the infrapyramidal blade (Figs. 4A,B, left). This may suggest that under normal circumstances, the higher levels of RA to which the infrapyramidal blade is exposed leads to decreased cell proliferation in comparison to that in the suprapyramidal blade. When mice were injected daily over a 3-week period with 13-*cis* RA the decline in proliferation induced by 13-*cis* RA treatment was primarily in the suprapyramidal blade (Fig. 4B, right). With the two blades exposed to equally high levels of RA this leads to normalization of the relative level of proliferation between the two blades. When the average ratio of BrdU labeling in the suprapyramidal to infrapyramidal blade was calculated, a ratio of 1.75 of suprapyramidal to infrapyramidal BrdU labeling was significantly reduced to a ratio of 0.91 following 13-*cis* RA treatment (Fig. 4C). This normalization of the two blades was also evident with shorter-term 13-*cis* RA treatment and when all-*trans* RA was used as the direct ligand for the RA receptor, rather than the prodrug (Fig. 4D). The well-established functional differences that exist along the septotemporal axis of the hippocampus (Moser and Moser,^1998^) include differences in neurogenesis in the dentate gyrus (Jinno,^2011^). The influence of all-*trans* RA to normalize the ratio of proliferating cells in the suprapyramidal versus infrapyramidal blade of differing subregions of the dentate gyrus was explored. Coronal sections along the rostral/caudal axis of the forebrain were divided into rostral, intermediate, and caudal regions, which sampled from different positions along the septotemporal axis of the hippocampus (Fig. 4E). It was found that the relative effect of all-*trans* RA to normalize the ratio between the two blades depended on the region examined and whereas the treatment had no effect in rostral regions, the effect was pronounced in intermediate and caudal hippocampus (Fig. 4E, i, ii, and iii), although this was only significant in the intermediate area.

**FIGURE 5:**
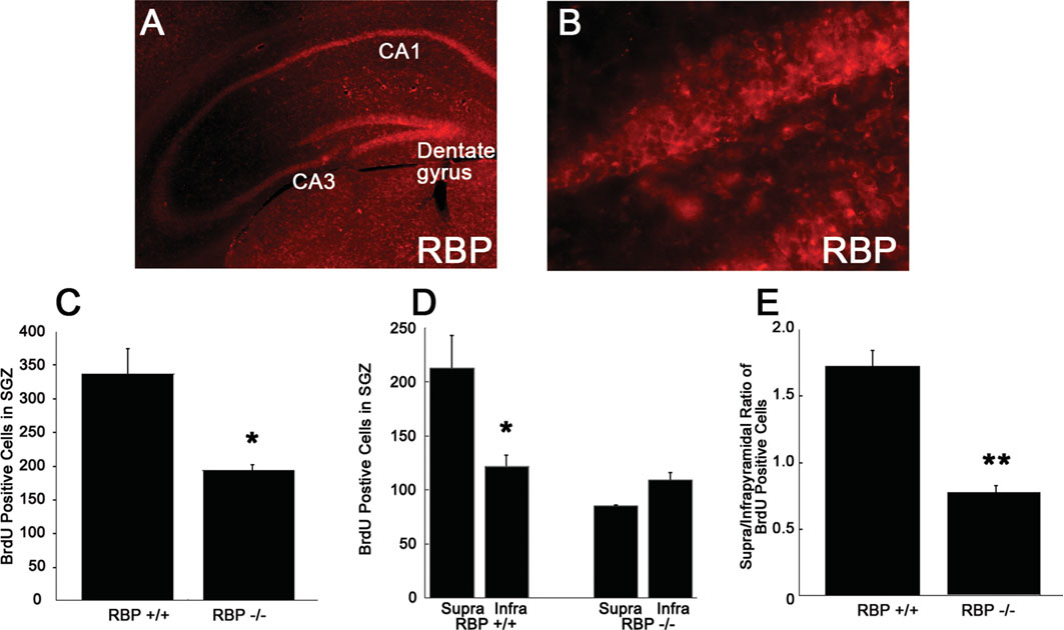
Expression of retinol binding protein (RBP) in the dentate gyrus and influence on cell proliferation. Immunohistochemistry for RBP indicated its presence in the hippocampus, including the dentate gyrus and CA1 and CA3 hippocampal subfields (A). A higher magnification view of the dentate gyrus indicates that RBP is present in the cytoplasm of cells throughout the dentate gyrus (B). The requirement of RBP for cell proliferation in the SGZ was examined by quantitation of BrdU-labeled cells, finding that cell proliferation was significantly lower in the RBP null mutant mouse compared with wild-type control (C). When this change is examined in the suprapyramidal versus infrapyramidal blades it is evident that almost all the decrease occurs in the suprapyramidal blade (D) and when measured as a ratio between the two blades the 1.7-fold difference between suprapyramidal versus infrapyramidal blades is significantly reduced to a 0.8-fold difference (E). Scale bars: A, 90 μm; B, 10 μm; DG, dentate gyrus; SGZ, subgranular zone. (C) Wild-type versus RBP null mutant, *n* = 3, Student's *t* test, *P* ≤ 0.02 (*), (D) RBP +/+ supra versus RBP +/+ infra, *n* = 3, one-way ANOVA, *P* < 0.01, Tukey's posthoc test, *P* < 0.05 (*), (E) wild-type versus RBP null mutant, *n* = 3, Student's *t* test, *P* ≤ 0.002 (**). [Color figure can be viewed in the online issue, which is available at wileyonlinelibrary.com.]

A genetic route by which retinoid signaling may be altered in the hippocampus is through mutation of the retinoid carrier protein retinol binding protein (RBP, also called RBP4). Systemically, RBP is synthesized by the liver and transports retinol from liver into the plasma to the target tissue requiring retinoids (Blaner,^1989^). Mice with RBP null mutations rely on retinoids carried by chylomicrons (Quadro et al.,^2003^) to support physiological retinoid-dependent functions and, aside from visual defects during the first months of life, the mutant mice show no obvious signs of deficiency in RA signaling when maintained on a retinoid sufficient diet. High levels of RBP mRNA are expressed in several forebrain regions, including the hippocampus (Komatsu et al.,^2005^). Immunostaining for RBP protein in the hippocampus detected immunoreactivity in both the dentate gyrus and CA1 and CA3 hippocampal subfields (Fig. 5A), with strong immunostaining in the cytoplasm of granule neurons (Fig. 5B). The function of RBP in the brain is unknown but RBP binds with equal affinity to retinol and RA (Breustedt et al.,^2006^) and cytoplasmic RBP may buffer retinoids and reduce the amount of RA entering the nucleus. Null mutation of RBP would thus increase RA transcriptional activation and decrease cell proliferation. Comparing the RBP null mutant with wild-type control, a significant decline in cell proliferation was evident, by 42% (Fig. 5C). When comparing the blades of the dentate gyrus, it was found that similar to the effects of RA treatment, almost all the decline in cell proliferation in the SGZ of the RBP null mutant animal occurred in the suprapyramidal blade (Fig. 5D), changing the ratio of BrdU labeling in the suprapyramidal to infrapyramidal blade from 1.7 to 0.8 in the RBP null mutant mouse (Fig. 5E).

**FIGURE 6:**
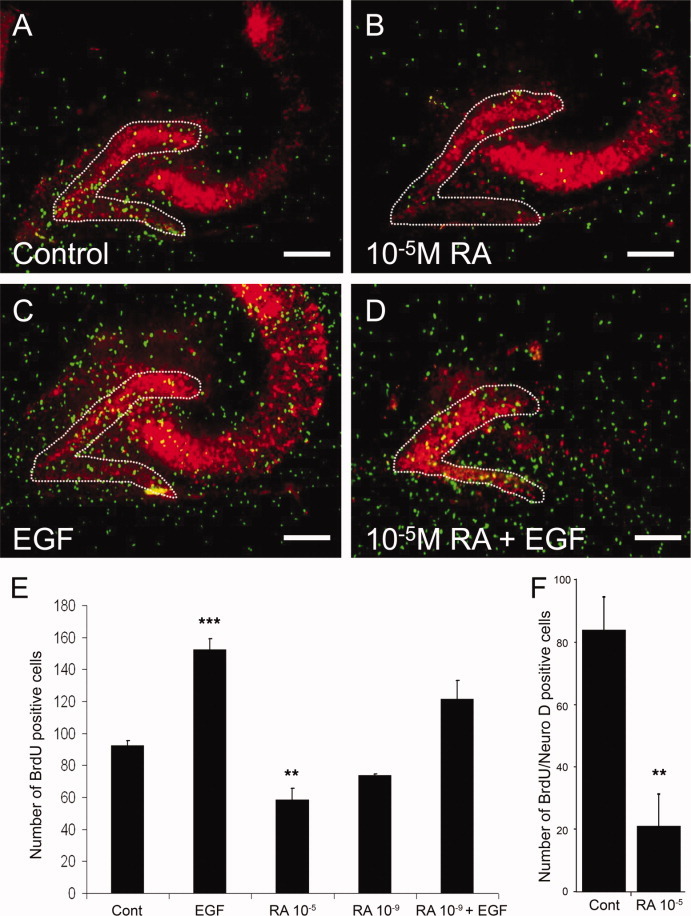
Influence of EGF and RA on cell proliferation in hippocampal slice cultures. BrdU (green) expression in a control-treated slice, double-labeled with NeuN (red); the dentate gyrus is outlined with a white dashed line as its position cannot be always clearly seen at the contrast used (A). Treatment with 10 μM RA significantly reduced proliferation in the dentate gyrus (B and E), whereas treatment with EGF significantly increased proliferation (C and E), and RA could partially suppress EGF's induction of proliferation (D and E). These changes in cell proliferation could be quantified by counting number of BrdU-labeled cells in the dentate gyrus following the different treatments (E). The influence of all-*trans* RA on neuronal precursors was determined by double-labeling for BrdU and the neuronal progenitor marker NeuroD, showing a strong and significant decrease in progenitor number. Scale bars: A-D, 200 μm*.* Control treated versus EGF treated, *n* = 6, one-way ANOVA, *P* < 0.0001, Tukey's posthoc test, *P* ≤ 0.001 (***). Control treated versus 10-5M RA treated, *n* = 6, one-way ANOVA, *P* ≤ 0.0001, Tukey's posthoc test, *P* < 0.01 (**) (F) control treated versus all-trans RA treated, *n* = 3–4, Student's *t* test, *P* ≤ 0.009 (**). [Color figure can be viewed in the online issue, which is available at wileyonlinelibrary.com.]

The inhibitory action of RA on cell proliferation in either the RA-treated mice or the RBP mutant is not necessarily directly on the hippocampus but may be a systemic action of RA influencing other growth regulatory factors or hormones. To investigate the effect of all-*trans* RA directly on cell proliferation in thehippocampus, organotypic slice cultures were used. Hippocampi from postnatal Day 14 mice were used because adult hippocampal slices do not survive a sufficient length of time to study cell proliferation. Hippocampal slices were cultured for 4 days after which regulatory factors were added for 2 days, BrdU added for a period of 2 hr and the slices fixed for immunohistochemistry. Proliferative cells in the SGZ can migrate within the dentate gyrus and so BrdU positive cells were counted throughout the dentate gyrus as previously described (Chechneva et al.,^2005^; Laskowski et al.,^2005^; Sadgrove et al.,^2006^; Namba et al.,^2007^). In these preparations, proliferating cells were evident both in the dentate gyrus as well as around the hippocampus, the latter presumably including glial cell progenitors (Fig. 6A). all-*trans* RA (10 μM) significantly inhibited cell proliferation in the dentate gyrus (Figs. 6B,E), whereas cell proliferation could be significantly promoted by EGF at 20 ng/ml (Figs. 6C,D). All-*trans* RA could limit this induction by EGF but this did not reach statistical significance (Figs. 6D,E). To determine the influence of all-*trans* RA specifically on neuronal precursors, the hippocampal slices were double-labeled for BrdU and the neuronal progenitor marker NeuroD. In comparison to vehicle-treated slices, this resulted in a significant fourfold decline in number of these progenitors. These results demonstrated that all-*trans* RA has predominantly an inhibitory action on hippocampal cell proliferation.

## DISCUSSION

**FIGURE 7:**
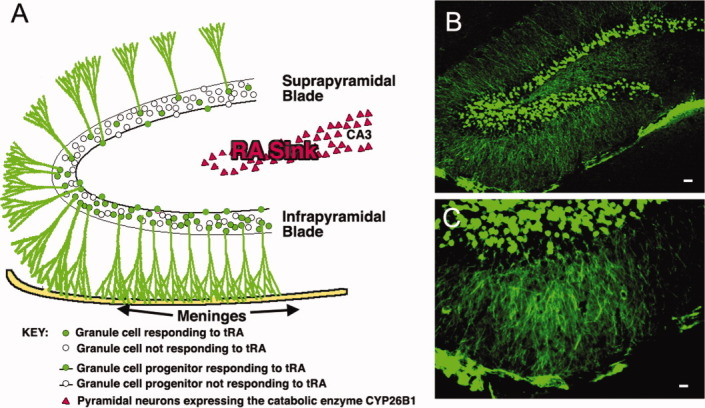
Model of RA signaling in the dentate gyrus in control animals. (A) RA is generated by RALDH2 in the meninges and activates RA signaling predominantly in granule cells and their precursors (in the SGZ) in the infrapyramidal blade of the dentate gyrus. Expression of the catabolic enzyme CYP26B1 further limits the diffusion of RA across the dentate gyrus. The dendrites of the granule neurons of the infrapyramidal blade stretch to reach the meninges, as can be seen in neurons immunofluorescently labeled for the RA reporter (B and C), as also illustrated in (A) and RA may hypothetically be transported by these processes to reach the granule neuron cell bodies. Scale bars: B, 50 μm; C, 25 μm. [Color figure can be viewed in the online issue, which is available at wileyonlinelibrary.com.]

In the developing CNS RA coordinates patterned gene expression via its own organization into regions of high and low concentration, these concentration differentials being determined by regional expression of synthetic and catabolic enzymes (McCaffery et al.,^1999^; Reijntjes et al.,^2004^; Hernandez et al.,^2007^) which are frequently in juxtaposition to each other (Swindell et al.,^1999^; Reijntjes et al.,^2005^). This report implies parallels between the adult and embryonic mouse brain with RA in the adult hippocampus distributed nonhomogenously across the dentate gyrus through localization of the synthetic and catabolic enzymes. The catabolic enzyme Cyp26b1 in cells of the hilus (Abu-Abed et al.,^2002^) provides a RA “sink” and may further accentuate the gradient of RA across the dentate gyrus. This is shown diagrammatically in Figure 7, illustrating RA synthesis by RALDH2 (and RALDH1) in the meninges, preferentially activating RA signal in cells of the infrapyramidal blade of the dentate gyrus. A lower amount of RA from a meningeal source would be expected to reach the suprapyramidal blade and RA for this blade may be provided from the low levels (1–2 nM) present in the circulation (Kurlandsky et al.,^1995^) and RALDH1 in the walls of large blood vessels (see Figs. 1A,B). In the embryonic hindbrain, RA can diffuse at least 200 μm (Smith et al.,^2001^), and thus, RA generated by the meninges could traverse the distance to the infrapyramidal blade. It may be considered though that the neuropil of the adult brain may be less open to diffusion, compared with the embryonic brain, and it is possible that RA, and perhaps other retinoids, are also actively transported into the granule cells of the infrapyramidal blade; the dendrites of the infrapyramidal granule neurons in the molecular layer coming in very close proximity to the meninges (Figs. 7B,C) and the retinoid binding proteins may capture RA. Evidence for the existence of this RA differential between blades is provided by the asymmetrical distribution of a RARE-lacz reporter in the two blades, showing greater number of reporter-labeled cells in the infrapyramidal compared with suprapyramidal blade; this difference could be ablated by a 3-day application of exogenous RA.

One potential result of this RA distribution is differential regulation of cell proliferation between the two blades of the dentate gyrus. In the untreated mouse, lower cell proliferation was evident in the infrapyramidal versus suprapyramidal SGZ, as determined by BrdU labeling. It may be conjectured that the lower cell proliferation in the infrapyramidal SGZ is due to the higher endogenous concentrations of RA around this blade. Cell proliferation in the suprapyramidal blade could be suppressed to similar levels by exogenous RA exposure either over an extended 3-week, or short 3-day time interval. It could not be determined from these results however, whether this was a direct or indirect influence of RA. Certainly, though RA has the potential to block cell division and does so in a number of progenitor cells (Clarke et al.,^2004^) and in organotypic slice culture, it was shown that RA can inhibit cell proliferation in the hippocampus. A marked difference in the capacity of RA to differentially effect proliferation in infrapyramidal versus suprapyramidal SGZ was evident between different locations along the forebrain rostral–caudal axis with rostral regions essentially nonresponsive. This presumably reflects the difference in the relative position along the septotemporal axis along which the hippocampus is both patterned in function (Moser and Moser,^1998^) and rates of neurogenesis (Jinno,^2011^). However, the mechanism that generates this difference in response is unknown and will require future explorations of differences in RA synthesis along this axis as well as the signaling system required to responds to RA, including the RA receptors and nuclear receptor coactivators and corepressors.

Mutation of RBP resulted in a similar reduction in the suprapyramidal blade as application of exogenous RA implying that loss of RBP may also lead to an increase in hippocampal RA signaling. RBP was first described as the liver synthesized binding protein that transports retinol in the circulation and it is essential, for instance, for the movement of retinol across the placenta into the embryo (Quadro et al.,^2005^). However, its expression in the brain is unlikely to equate with such a role because the enzymes necessary for oxidation of retinol to active RA are present in very few regions of the brain (Wagner et al.,^2002^). It had been previously presumed that RBP was not important for the brain given that RA does not cross the blood brain barrier (Breustedt et al.,^2006^); however, RBP is extensively expressed within the brain of both the mouse (unpublished finding) and primate (Komatsu et al.,^2005^). In the brain the affinity of this protein for RA, previously not considered relevant because RA levels in the plasma are very low (Breustedt et al.,^2006^), may be of importance for its function in the brain. That the RBP null mutant resulted in a decline in proliferation in a similar direction to that of excess RA suggests that RBP may diminish the action of RA and a decline in this binding protein increases RA signaling. RBP may buffer RA or it is conceivable that RBP may have the function of secreting retinoids from neural cells, in a similar way to that RBP secretion from the liver is combined with the release of retinol (Ronne et al.,^1983^). A question to resolve is its function relative to CRABPI, which has a 10-fold higher affinity for RA than RBP (Fiorella and Napoli,^1991^; Breustedt et al.,^2006^), although the relative amounts of RBP and CRABPI are unknown. A difference may exist in the subcellular distribution of the proteins, for instance, RBP is associated with the Golgi complex and secretory vesicles (Suhara et al.,^1990^), whereas CRABPI is present in a perinuclear location (Levadoux-Martin et al.,^2006^) and is also transported into the nucleus (Gaub et al.,^1998^); their cellular localization will influence the fraction of RA bound by both. It will be of interest to determine whether CRABPI null mutations in the mouse, which appear essentially normal (Gorry et al.,^1994^), show an effect on hippocampal cell proliferation.

RA-mediated differences between the infrapyramidal and suprapyramidal blades may regulate other processes in the blades in addition to cell proliferation. Although most aspects of dentate gyrus function are identical between the blades, varied features distinguishing them have been identified. Granule cell differences are evident in dendrite length (Claiborne et al.,^1990^) as well density of their spines (Desmond and Levy,^1985^). Differences in cell vulnerabilities exist; for instance, IL-2 deficiency results in a greater loss of infrapyramidal than suprapyramidal granule cell numbers (Beck et al.,^2005^), whereas the infrapyramidal blade shows a greater sensitivity to hypoxia (Hara et al., 1990). Mutation of Lmx1a in the Dreher mouse preferentially results in loss of the infrapyramidal blade (Sekiguchi et al.,^1992^), whereas mutation of EphB2 results in loss of just the lateral portion of the suprapyramidal blade (Catchpole and Henkemeyer,^2011^). Numbers of neurons can differ between the blades, for instance, in two strains of seizure-prone mice numbers of granule and basket cells were higher in the suprapyramidal than infrapyramidal blade (Wimer et al.,^1990^). Granule cell activity in the suprapyramidal blade has recently been found to be higher than in the infrapyramidal blade as determined by the expression of the immediate early gene zif268 (Schmidt et al.,^2012^), and this same report lists numerous examples of connectivity differences. The source of RA, from RALDHs in the meninges, also provides RA for the developing CNS (Zhang et al.,^2003^) and, for instance, meningeal RA regulates forebrain development (Siegenthaler et al.,^2009^). RA may be among the factors synthesized by the meninges necessary for the postnatal development of the infrapyramidal blade given that removal of the meninges results in failure of this blade to develop (Hartmann et al.,^1992^).

In contrast to the influence found in this study of exogenous RA applied in vivo or in vitro to inhibit proliferation, the studies of Jacobs et al. (Jacobs et al.,^2006^) have shown that vitamin A in the mouse is necessary for neuronal differentiation in the SGZ but no effect was evident on cell proliferation. Our previous findings (Crandall et al.,^2004^; Sakai et al.,^2004^) as well as those of others (Jung et al.,^2007^), found that exposure to RA can inhibit cell proliferation (and neurogenesis) in the SGZ or the SVZ. It is possible that these differences may, in part, reflect the precursor type examined; Jacobs et al. studied precursors labeled following exposure to BrdU over a 6-day period as opposed to the much briefer 6 hr exposure to BrdU used in this study. Surprisingly, all the parameters of neurogenesis measured by Jacob et al. (2006) in the hippocampus which were altered as a result of vitamin A deficiency, were not reversible by the addition of RA, suggesting in these studies some additional, RA independent, roles for retinol.

The few reports that have examined the relative differences in neurogenesis between the blades of the dentate gyrus have been conflicting regarding relative levels of, for instance, survival. It has been reported that survival of BrdU-labeled cells is higher in the suprapyramidal versus infrapyramidal (Ambrogini et al.,^2000^) whereas a more recent study has indicated the opposite result when blades are compared (Snyder et al.,^2009^) which disappears with age (Snyder et al.,^2011^). These differences may be due to the varied survival time being measured (15 vs. 28 days) or strain differences. The consequences for hippocampal function of such differences are presently unknown. However, a detailed study by Jinno (2011) of spatial differences in neurogenesis found greater number of calretinin-positive cells, a marker of immature granule cells, in the suprapyramidal versus infrapyramidal blade of the dorsal dentate gyrus, where calretinin-positive cells reside. Based on the findings of Jinno (2011) that neurogenesis is patterned both in a dorsal/ventral and suprapyramidal/infrapyramidal blade orientation, and that hippocampal neural interconnectedness and function show difference in this organization, it was proposed “that the topographic organization of the neural circuits might contribute to these interblade differences in adult neurogenesis.”

In summary, this study has identified an asymmetry in RA signaling between the suprapyramidal and infrapyramidal blades of the dentate gyrus and this difference may be driven by a gradient of RA across the dentate gyrus from the local source of RALDH1 and RALDH2 in the meninges. Such a gradient parallels its action as a morphogen in the embryo and may help regulate those processes found to be dissimilar between the two blades. It cannot be excluded that the relative difference in effect of RA on the suprapyramidal and infrapyramidal blades reflects an intrinsic difference in RA signaling between cells of the two blades. Aside though from the asymmetrical distribution of the RALDHs, no other protein that mediates RA signaling was found to be different between the two blades, including RARbeta, RARgamma, CRABPI, and RBP. Similarly, it is possible that the difference in cell proliferation between the two blades may involve factors integral to the two blades. Given though the findings that both exogenous RA and mutation of RBP ablate the difference between the two blades, and that in organotypic slice cultures, RA inhibits cell proliferation, the simplest explanation is that the difference is due to the existence of an asymmetry in RA concentration. This hypothesis will be further tested in the future via manipulation of this putative disparity by null mutation of other genes necessary for RA signaling.
